# 2-phenylethynesulfonamide Prevents Induction of Pro-inflammatory Factors and Attenuates LPS-induced Liver Injury by Targeting NHE1-Hsp70 Complex in Mice

**DOI:** 10.1371/journal.pone.0067582

**Published:** 2013-06-21

**Authors:** Chao Huang, Jia Wang, Zhuo Chen, Yuzhe Wang, Wei Zhang

**Affiliations:** 1 Department of Pharmacology, School of Medicine, Nantong University, Nantong, Jiangsu, People’s Republic of China; 2 Invasive Technology Department, Nantog First People‚s Hospital, Nantong, Jiangsu, People’s Republic of China; French National Centre for Scientific Research, France

## Abstract

The endotoxin-mediated production of pro-inflammatory cytokines plays an important role in the pathogenesis of liver disorders. Heat shock protein (Hsp70) overexpression has established functions in lipopolysaccharide (LPS)-mediated inflammatory response. However, little is known about the role of Hsp70 activity in LPS signaling. We hypothesized that inhibition of Hsp70 substrate binding activity can ameliorate LPS-induced liver injury by decreasing induction of pro-inflammatory factors. In this study, C57/BL6 mice were injected intraperitoneally with LPS and 2-phenylethynesulfonamide (PES), an inhibitor of Hsp70 substrate binding activity. We found that i. PES prevented LPS-induced increase in serum alanine aminotransferase (ALT) and aspartate aminotransferase (AST) activity, infiltration of inflammatory cells, and liver cell apoptosis; ii. PES reduced inducible nitric oxide synthase (iNOS) protein expression as well as serum nitric oxide (NO), tumor necrosis factor-α (TNF-α), and interleukin-6 (IL-6) content in LPS-stimulated mice; iii. PES reduced the mRNA level of iNOS, TNF-α, and IL-6 in LPS-stimulated liver. iiii. PES attenuated the degradation of inhibitor of κB-α (IκB-α) as well as the phosphorylation and nuclear translocation of nuclear factor-κB (NF-κB) in LPS-stimulated liver. Similar changes in the protein expression of inflammatory markers, IκB-α degradation, and NF-κB phosphorylation and nuclear translocation were observed in RAW 264.7 cells. Further mechanistic studies revealed that PES remarkably reduced the elevation of [Ca^2+^]_i_ and intracellular pH value (pH_i_) in LPS-stimulated RAW 264.7 cells. Furthermore, PES significantly reduced the increase in Na^+^/H^+^ exchanger 1 (NHE1) association to Hsp70 in LPS-stimulated macrophages and liver, suggesting that NHE1-Hsp70 interaction is required for the involvement of NHE1 in the inflammation response. In conclusion, inhibition of Hsp70 substrate binding activity *in vivo* reduces the induction of pro-inflammatory factors and prevents LPS-induced liver injury likely by disrupting NHE1-Hsp70 interaction which consequently reduces the activation of IκB-α-NF-κB pathway in liver.

## Introduction

The importance of macrophage activation and endotoxin-mediated induction of pro-inflammatory factors in liver injury is evident from numerous models of liver disorders. For example, one previous study showed that lipopolysaccharide (LPS) triggers liver injury by elevating tumor necrosis factor-α (TNF-α) release in a non-alcoholic steatohepatitis (NASH) model [1]. In a mouse model of intestinal injury, intestinal-derived LPS mediates liver injury by increasing TNF-α and interleukin-6 (IL-6) release through Toll-like receptor activation [2]. In ischemia-reperfusion liver injury, LPS also promotes the induction of pro-inflammatory cytokines such as TNF-α, IL-6, and IL-1β [3]. Thus exposure to LPS induces inflammation induction, contributing to the development of liver injury. Interfering with the LPS-induced inflammatory response would then be beneficial to cope with the inflammation associated with liver disorders.

Heat shock protein 70 (Hsp70) is a highly conserved stress-induced protein. It contains a conserved N-terminal ATPase domain and a variable C-terminal substrate binding domain [4]. Its involvement in LPS-mediated inflammatory responses has been reported. For instance, overexpression of Hsp70 was found to suppress pro-inflammatory factors induction in macrophage [5] and microglia [6], and to favor human liver recovery from ischemia-reperfusion by preventing LPS-induced nuclear factor kappa B (NF-κB) activation [7]. The role of Hsp70 substrate binding activity in LPS signaling is yet unknown. Recently, an inhibitor of Hsp70 substrate binding activity, 2-phenylethynesulfonamide (PES), has been found to promote cancer cell death [8]. Data from human bone marrow leukemic blasts also suggests that Hsp70 substrate binding activity inhibition is an effective strategy to against leukemia [9]. Here we investigated whether PES could interfere with the LPS signaling.

The cellular actions of Hsp70 are mediated mainly by its physical association with a number of co-chaperones. For example, Hsp70 was reported to associate with APAF1, an important apoptosis regulator, to modulate apoptosome formation [10], [11], [12]. In binding to Hsp70, C terminus of Hsc70-interactiong protein (CHIP) and BAG-1 may serve as a link between the chaperone and the proteasome, perhaps aiding in the targeting of substrates for degradation [13], [14], [15]. PES has been confirmed to diminish the interaction of Hsp70 with different molecules, including APAF1, CHIP, and BAG-1 [8]. Because Na^+^/H^+^ exchanger 1 (NHE1) is considered as a mediator of inflammation response [16] in macrophages and has been reported to interact with Hsp70 [17], we hypothesized that PES would exert an anti-inflammatory function by reducing the LPS-induced increase in NHE1-Hsp70 association in liver. To test this, we examined the effect of inhibiting Hsp70 substrate binding activity *in vivo* using PES on LPS-mediated liver injury and induction of pro-inflammatory factors in mice. To dissect the molecular mechanism underlying the inhibition of induction of pro-inflammatory factors by PES, we performed *in vitro* experiments in cultured macrophages. Here, we found that inhibition of Hsp70 substrate binding activity protects against LPS-mediated liver injury by suppressing induction of pro-inflammatory factors likely through reducing degradation of inhibitor of κB-α (IκB-α) and activation of NF-κB in liver. Our data also provides direct evidence for the indispensable role of NHE1-Hsp70 association in the generation of liver inflammatory response. Thus strategies that disrupt the NHE1-Hsp70 association in liver might be beneficial in treating acute liver inflammation.

## Materials and Methods

### Chemical and Reagents

PES and BAPTA-AM were purchased from Calbiochem (San Diego, CA, USA). LPS was the product of Sigma (Saint Louis, MO, USA). Fura-2 and BCECF-AM were obtained from Biotium (Hayward, CA, USA). Antibodies against Histone H2A, glyceraldehyde-3-phosphate dehydrogenase (GAPDH), inducible nitric oxide synthase (iNOS), IκB-α, p-NF-κB p65 (Ser536), and NF-κB p65 were purchased from Cell Signaling Technology (Beverly, MA, USA). Antibody against NHE1 was the product of Millipore (Billerica, MA, USA). Protein A/G PLUS-Agarose was the product of Santa Cruz Biotechnology (Santa Cruz, CA, USA). Other related agents were purchased from commercial suppliers. PES was dissolved in dimethylsulfoxide (DMSO). The final concentration of DMSO was <0.05%. No detectable effects of DMSO were found in our experiments. All drugs were prepared as stock solutions, and stock solutions were stored at –20°C.

### Animals and Experimental Protocol

The use of C57BL/6 male mice was approved by the University Animal Ethics Committee of Nantong University (Permit Number: 2110836). 6–8-week old mice were randomly divided into four groups. 0.25 mg/kg body weight (BW) of LPS was selected to induce inflammation according to previous studies [18], [19]. In saline and LPS-treated groups, mice were injected intraperitoneally with 100 µL of saline and 0.25 mg/kg LPS in 100 µL of saline. In PES alone-treated groups, mice were administered a single dose of PES at 1, 5, and 10 mg/kg doses. In PES and LPS-co-treated groups, mice were administered a single dose of PES at 1, 5, and 10 mg/kg doses 1 h before LPS injection. After 2 h of LPS injection, the whole blood was collected from ophthalmic vein and kept at room temperature for 30 min. Followed by centrifugation at 2000 *g* for 15 min, the supernatants were transferred into a new tube and frozen at –80°C. After that, the whole livers were immediately excised and a portion was snap-frozen in liquid nitrogen and stored at –80°C. Additional portions of the liver were stored in RNA stabilization reagent, RNAlater (Qiagen GmbH, Hilden, Germany), for RNA extraction.

### Cell Culture

RAW 264.7 cells were purchased from ATCC (Manassas, VA, USA) and grown in DMEM/F12 with 10% fetal bovine serum (FBS) (Gibco). The medium was replaced every 3 days. All cells were grown in 37°C incubator containing 95% air and 5% CO_2_. After treatment, cell supernatants were collected and frozen at –80°C for NO detection.

### Cell Viability Assay

Cell viability was measured using MTT Cell Proliferation and Cytotoxicity Assay Kit (Bi Yuntian Biological Technology Institution, Shanghai, China). Briefly, methylthiazolyldiphenyl-tetrazolium bromide (5 mg/mL) was dissolved in prepared MTT-dissolved solutions and kept at –20°C. After washing with PBS, the cells in plates were added 20 µL of MTT solutions and kept at 37°C for 4 h. The blue crystals were dissolved in formazan-dissolved solutions. The absorbance was read at 570 nm.

### Serum Biochemical Assay and Cytokines Measurement

Serum alanine aminotransferase (ALT) activity and aspartate aminotransferase (AST) activity were determined using the colorimetric assay kits from Jianchen Biology Engineering Institute (Nanjing, China). Serum TNF-α and IL-6 levels were determined using cytokine specific BD OptEIA enzyme-linked immunosorbent assay kits (BD Biosciences Pharmingen, San Diego, CA).

### Hematoxylin and Eosin (HE) Staining and Apoptosis Assay

To prepare liver slices, mice from different groups were deeply anesthetized with sodium pentobarbital (60 mg/kg) and then rapidly perfused with warm (37°C) sailne and 4% paraformaldehyde in 0.01 M phosphate buffer (pH 7.4). Livers were then removed and post-fixed in 4% paraformaldehyde for 4 h. Following transfer to 30% surose solution overnight at 4°C, the livers were chipped into 20 µm thickness slices with a freezing microtome (CM1900, Leica Microsystem). For HE staining, slices were incubated with HE solutions (Bi Yuntian Biological Technology Institution, Shanghai, China). The apoptosis cells in liver were detected with one step terminal deoxynucleotidyl transferase dUTP nick end labeling (TUNEL) assay kit from Bi Yuntian Biological Technology Institution (Shanghai, China). The images of HE staining and TUNEL-positive cells were viewed with a Nikon Eclipse 800 microscope.

### NO Detection

Total nitrite levels were measured with a Griess reagent kit (Invitrogen). The reaction consisted of 20 µL of Griess Reagent, 150 µL of serum or cell supernatants, and 130 µL of de-ionized water. After incubation of the mixture for 30 min at room temperature, nitrite levels were measured at 548 nm using an M2 spectrophotometric microplate reader (Molecular Devices).

### Real-time PCR

At the end of each treatment, total RNA was isolated from livers or RAW 264.7 cells using the RNeasy mini kit according to the manufacturer’s instructions (Qiagen, GmbH, Hilden, Germany). First-strand cDNA was generated by reverse transcription of total RNA using the RT system (Promega, Madison, WI, USA). Real-time PCR reactions were conducted with Faststart SYBR Green Master Mix (Roche Molecular Biochemicals). Briefly, 2 µL of diluted cDNA, 0.5 µM primers, 2 mM MgCl_2_, and 1×FastStart SYBR Green Master mix were employed. The primers were quoted as follows [20], [21]: iNOS 5′-CTC ACT GGG ACA GCA CAG AA-3′ (forward), 5′-TGG TCA AAC TCT TGG GGT TC-3′ (reverse); TNF-α 5′-CAC CAC CAT CAA GGA CTC AA-3′ (forward), 5′-AGG CAA CCT GAC CAC TCT CC-3′ (reverse); IL-6 5′-ACA ACC ACG GCC TTC CCT ACT T-3′ (forward), 5′-CAC GAT TTC CCA GAG AAC ATG TG-3′ (reverse); 18S rRNA 5′-GTA ACC CGT TGA ACC CCA TT-3′ (forward), 5′-CCA TCC AAT CGG TAG TAG CG-3′ (reverse). PCR products were detected by monitoring the fluorescence increase of double-stranded DNA-binding dye SYBR Green during amplification. The expression levels of target genes were normalized to the house-keeping gene (18S rRNA). The fold-changes in the target gene expression between experimental groups were expressed as a ratio. Relative gene expression was calculated by the comparative cycle threshold (Ct) method. Melt-curve analysis and agarose gel electrophoresis were used to examine the authenticity of the PCR products.

### NF-κB binding assays

The nuclei were extracted from RAW 264.7 cells or liver tissues by firstly incubating them in hypotonic buffer (10 mM Tris-HCl, pH 7.5, 10 mM NaCl, 1.5 mM MgCl_2_·6H_2_O) at 4°C for 20 min. After homogenization, cell homogenates were spun at 3000 g for 5 min. The supernatants were collected for Western blot. The pellets were recovered, extensively washed, and re-suspended in the nuclear extraction buffer (50 mM Tris-HCl, pH 7.4, 150 mM NaCl, 1% Nonidet P-40, 0.25% sodium deoxycholate, 10% glycerol, 50 mM NaF, 1 mM Na_3_VO_4_, 5 mM sodium pyrophosphate, protease inhibitors). The NF-κB binding activity of nuclear extracts was measured with the TransFactor NF-κB colorimetric kit (Clontech, Mountain View) according to the manufacturer’s instruction.

### Western blot

To extract the total proteins, livers or cells were lysed on ice for 30 min in lyses buffer (50 mM Tris-HCl, pH 7.4, 1 mM EDTA, 100 mM NaCl, 20 mM NaF, 3 mM Na_3_VO_4_, 1 mM PMSF, with 1% (v/v) Nonidet P-40, and protease inhibitor cocktail) [22], [23]. The lysates were centrifuged at 12000 *g* for 15 min, and the supernatant were recovered. After denaturation, 50 µg of proteins were separated on 10% SDS/PAGE gels and then transferred to nitrocellulose membranes by using a transfer cell system (Bio-Rad, California, USA). After blocking with 5% nonfat dried milk powder/Tris-buffered saline Tween-20 for 1 h, membranes were probed with 1:500 primary antibodies against iNOS, IκB-α, p-NF-κB, NF-κB, and Histone H2A or 1∶10000 primary antibody against GAPDH overnight at 4°C. Primary antibodies were then removed by washing the membranes 3 times in TBST, and incubated for further 2 h at room temperature with IRDye 680-labled secondary antibodies (1∶3000–1∶5000). Immunoblots were visualized by scanning using Odyssey CLx western blot detection system. For isolation of proteins in the cytoplasm and nucleus, Nucleus Protein Extraction kit was used according to supplier’s recommendations (Bi Yuntian Biological Technology Institution, Shanghai, China). Proteins in the cytoplasm and nucleus were checked by Western blot and were normalized to GAPDH and Histone-H2A, respectively. The band density was quantified using Image J software.

### Intracellular Ca^2+^ Measurement

RAW 264.7 cells grown on glass coverslips were washed three times with extracellular solution containing 150 mM NaCl, 5 mM KCl, 1 mM MgCl_2_·6H_2_O, 2 mM CaCl_2_, 1 mM glucose, 10 mM HEPES (pH 7.4) and incubated with 1 µM Fura-2/AM for 40 min at 37°C. Coverslips with Fura-2/AM-loaded cells were then mounted on a chamber positioned on the movable stage of an inverted microscope (Olympus IX70, Tokyo, Japan), which is equipped with a calcium imaging system (TILL Photonics). Fluorescence was excited at wavelengths of 340 nm for 150 ms and 380 nm for 50 ms at 1-s intervals by a monochromator (Polychrome IV), and the emitted light was imaged at 510 nm by an intensified cooled charge coupled device (TILL Photonics Image) through an X-70 fluor oil immersion lens (Olympus, Tokyo, Japan) and a 460 nm long-pass barrier filter. F340/F380 fluorescence ratio was recorded and analyzed with TILLVISION 4.0 software, which was used as an indicator of [Ca^2+^]_i_ independent of intracellular Fura-2 concentration. The amplitude of [Ca^2+^]_i_ response was defined as the peak of △F/F. All experiments were repeated at least three times using different batches of cells.

### Intracellular pH value (pH_i_) Measurement

For pH_i_ measurement, RAW 264.7 cells grown on coverlips were incubated with 2 µM BCECF-AM at 37°C for 30 min. The coverlips were placed in a temperature-controlled (37°C) open-bath imaging chamber containing HCO3^−^ free HEPES-MEM (model RC24; Warner Instruments). The chamber was mounted on the stage of the Olympus inverted epifluorescence at 535 nm was recorded. The ratio of the background-corrected fluorescence emissions (F_490_/F_440_) was calibrated using the high K^+^/nigericin techinique.

### Co-immunoprecipitation

The liver tissues or RAW 264.7 cells were harvested and lysed on ice for 40 min in normal lyses buffer. The lysates were then centrifuged at 12,000 *g* for 15 min, and the supernatants were recovered. Supernatants containing equal amounts of proteins were incubated with primary anti-Hsp70 antibody (1∶200) overnight (15 h) at 4°C. The Protein A/G PLUS-Agarose (40 µL in 50% slurry) was washed twice with regular washing buffer (50 mM Tris-HCl, 100 mM NaCl, 1 mM EDTA, and 0.5% Nonidet P-40), and then added into proteins for 2 h incubation at room temperature. At last, the mixture was washed five times with high salt washing buffer (50 mM Tris-HCl, 500 mM NaCl, 1 mM EDTA, and 0.5% Nonidet P-40). Pulled down proteins were boiled at 95°C for 5 min in SDS loading buffer and analyzed by Western blot.

### Statistical Analysis

Data were expressed as means ± SE. One-way ANOVA followed by subsequent post hoc analysis was used for the statistical analysis by employing SPSS 11.0 software. Differences were considered significant at *P*<0.05 or *P*<0.01.

## Results

### PES, an Hsp70 substrate binding activity Inhibitor, reduces serum ALT/AST levels and extent of apoptosis in LPS-stimulated liver

**Figure 1:**
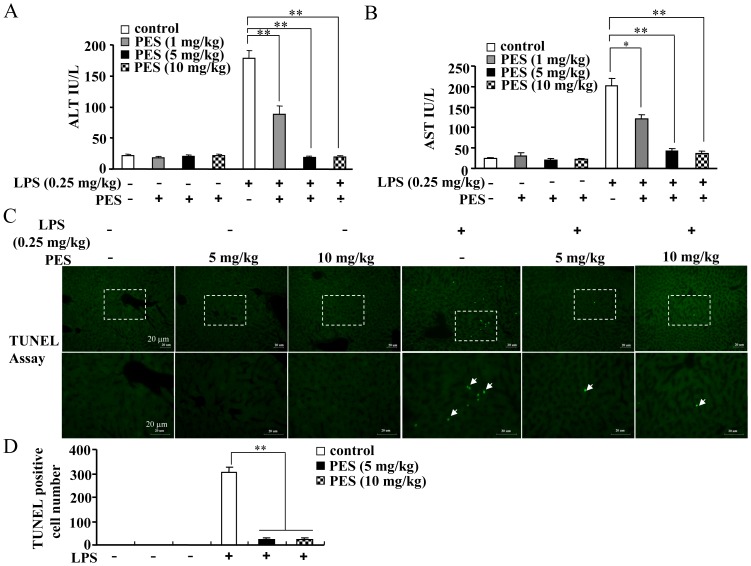
PES reduces LPS-induced increase in serum ALT/AST activity and extent of apoptosis in the liver. LPS (0.25 mg/kg) and PES (1, 5, and 10 mg/kg) were injected intraperitoneally in C57BL/6 mice. All mice were sacrificed 8 h post injection. Serum ALT (A) and AST (B) activity as well as the extent of apoptosis (C, D) were measured as described in methods. Values were shown as mean ± SE, n = 8, **P<0.01 vs. LPS alone-injected mice. The upper images in C were magnified at 200×. The boxed areas were magnified at 400×. Bar: 20 µm. Arrows: TUNEL-positive cells.

The significance of Hsp70 substrate binding activity in liver inflammatory response is not well understood. Here, we determined the *in vivo* effect of PES, an inhibitor of Hsp70 substrate binding activity, on LPS-mediated acute liver inflammatory injury. The levels of ALT and AST, markers of liver injury, were measured after 8 h of LPS and PES administration *in vivo*. Fig. 1A and 1B show that LPS injection (100 µL, intraperitoneally) *in vivo* at 0.25 mg/kg BW significantly elevated serum ALT and AST levels as compared to saline control, after 8 h. PES, administered at 1, 5, and 10 mg/kg BW, exhibited a dose-dependent reduction in serum ALT and AST levels. The peak change was observed at 5 and 10 mg/kg doses. In accordance with the change in serum ALT and AST levels, we observed a significant increase in the number of TUNEL-positive cells after LPS exposure (Fig. 1C, 1D), and the number of TUNEL-positive cells was remarkably reduced by PES treatment at 5 and 10 mg/kg doses (Fig. 1C, 1D). In the PES alone-treated groups, we did not find any significant changes of ALT/AST levels and the number of TUNEL-positive cells (Fig. 1). In general, inhibition of Hsp70 substrate binding activity ameliorated LPS-induced liver injury. Because administration of PES at 5 and 10 mg/kg shows similar effects, subsequent *in vivo* experiments were performed at 1 and 5 mg/kg.

### PES reduces the infiltration of inflammatory cells in liver

**Figure 2:**
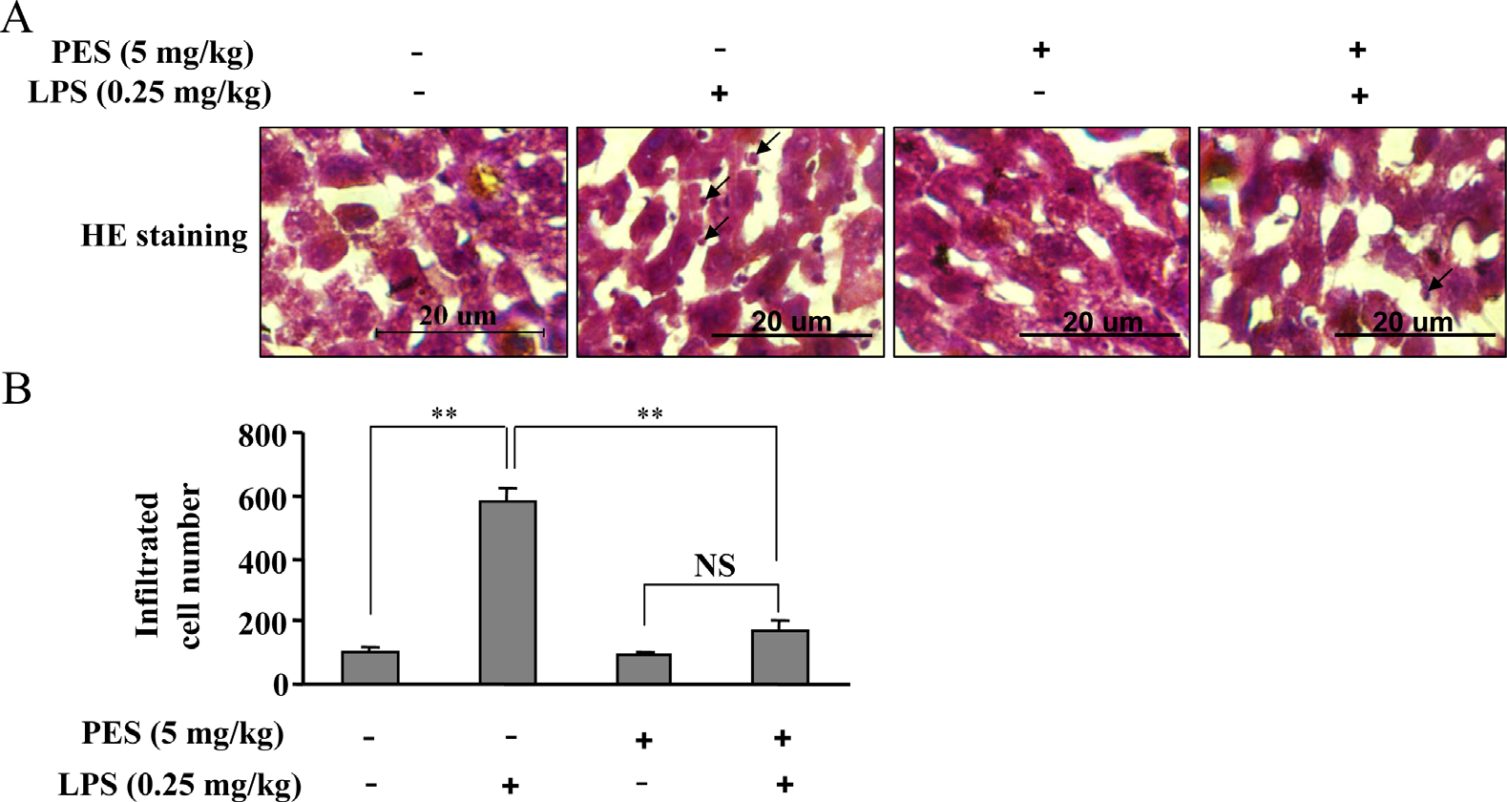
PES reduces the infiltration of inflammatory cells and iNOS induction in inflammatory cells. (A) Representative images showing that PES significantly reduced the infiltration of inflammatory cells in LPS-stimulated (0.25 mg/kg) liver. The HE images were magnified at 400×. Bar: 20 µm. Arrows: inflammatory cells. (B) Quantitative analysis of PES on the number of infiltrated cells in liver after LPS injection, n = 6, **P<0.01 vs. control. All data were shown as mean ± SE. NS: no significance.

LPS-induced liver injury is considered to be mediated by inflammatory factors, and these factors are synthesized primarily by inflammatory cells recruited to liver during inflammation [21]. Thus we assayed the morphological changes in liver tissue using HE staining after LPS and/or PES administration. Interestingly, we found that LPS stimulation (0.25 mg/kg, 8 h) induced a robust increase in the infiltration of inflammatory cells (Fig. 2A). PES, administered at 5 mg/kg, remarkably reduced this infiltration (Fig. 2A, 2B), indicating that the increase in inflammatory cells during acute LPS exposure might contribute to the LPS-induced injury of liver.

### PES reduces induction of pro-inflammatory factors in the liver

**Figure 3:**
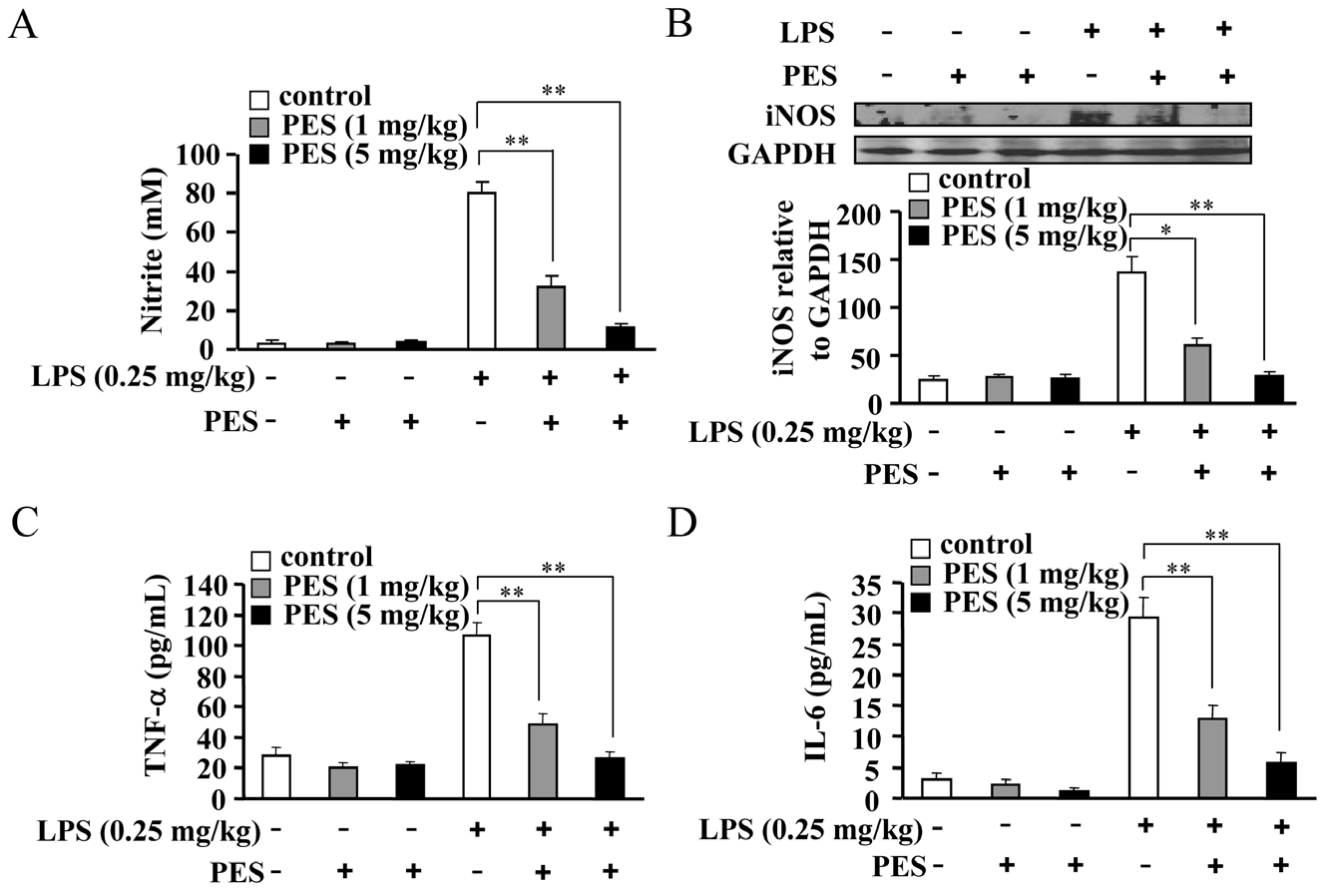
PES reduces LPS-induced increase in pro-inflammatory factors in liver. (A) Statistical data showing that pretreatment with PES (1 and 5 mg/kg, 1 h) reduced serum nitrite content after LPS injection (0.25 mg/kg), n = 8, **P<0.01 vs. LPS alone-injected mice. (B) The expression (upper) and quantitative analysis (lower) of iNOS in LPS/PES-injected liver, n = 6, *P<0.05, **P<0.01 vs. LPS alone-injected mice. (C, D) Quantitative analysis of PES on serum TNF-α (C) and IL-6 (D) content after LPS injection, n = 8, **P<0.01 vs. LPS alone-injected mice. All data were shown as mean ± SE.

**Figure 4:**
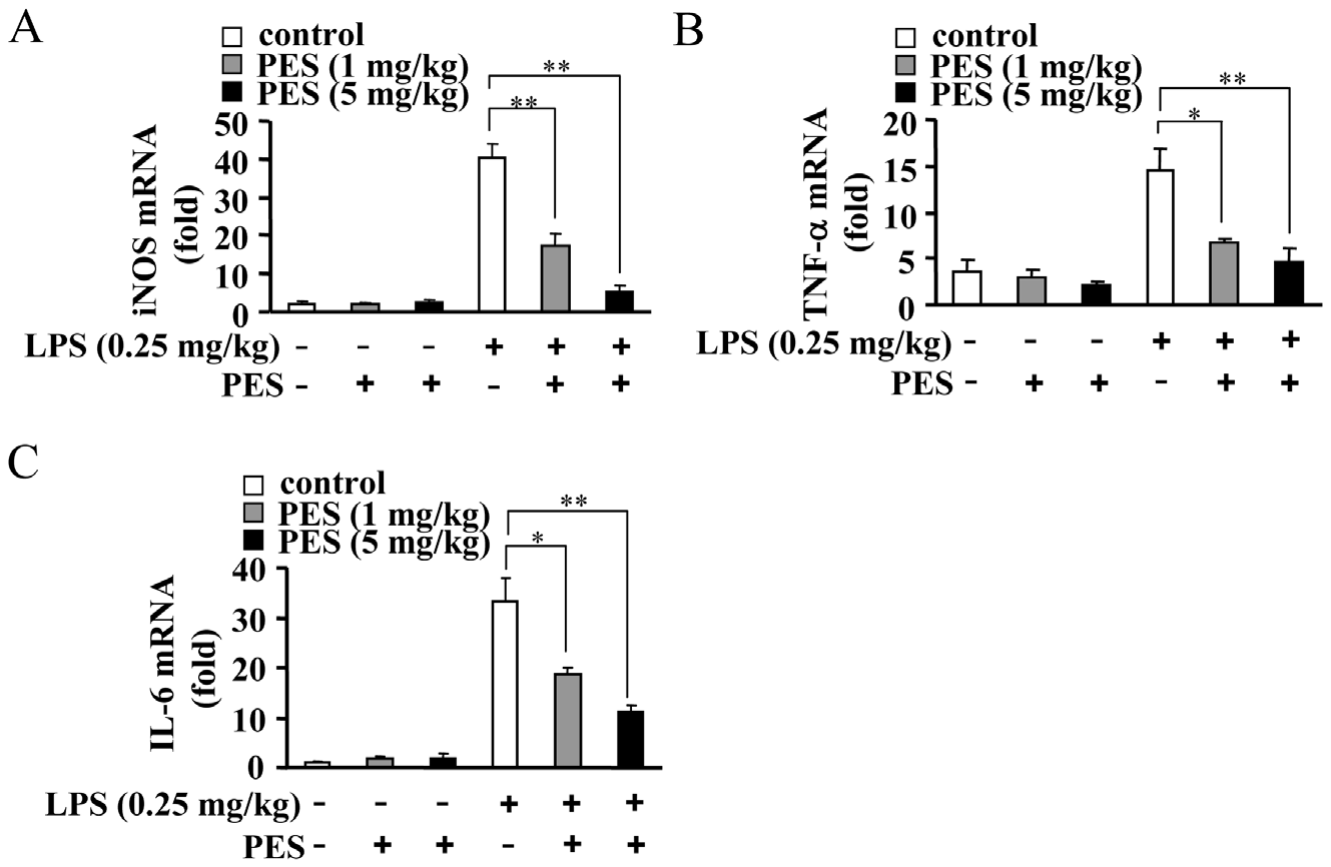
PES attenuates LPS-induced pro-inflammatory gene transcription in liver. C57BL/6 mice were injected intraperitoneally with LPS (0.25 mg/kg) and PES (1 and 5 mg/kg). Total RNA was extracted from liver tissues. Messenger RNA levels of liver iNOS (A), TNF-α (B), and IL-6 (C) were analyzed by quantitative real-time PCR, and normalized to 18S rRNA. Results were expressed as mean fold change ± SE over mice injected with saline, n = 6, *P<0.05, **P<0.01 vs. LPS alone-injected mice.

To further investigate whether PES could inhibit inflammation *in vivo*, we measured the changes in pro-inflammatory factors level in LPS- and/or PES-stimulated mice. First, we monitored the NO content in serum collected from mice treated with LPS and/or PES *in vivo*. As shown in Fig. 3A, serum NO content was significantly reduced by PES administration at 1 and 5 mg/kg BW, compared to LPS alone (0.25 mg/kg). Second, we monitored the change in iNOS expression in the whole liver after treatment with PES *in vivo*. As shown in Fig. 3B and 3C, a significant reduction in iNOS expression was observed at 1 and 5 mg/kg of PES treatment, compared to LPS alone, in the liver (Fig. 3B). Third, we measured the level of serum cytokines in mice sacrificed at 2 h after LPS and/or PES injection. We observed that TNF-α and IL-6 levels were significantly reduced by 1 and 5 mg/kg of PES, compared to LPS alone (Fig. 3C, 3D). The reduction in the expression of pro-inflammatory proteins could be due to the suppression of either gene transcription or protein translation. To differentiate between the two possibilities, we monitored iNOS, TNF-α, and IL-6 mRNA levels in LPS-treated liver in the presence or absence of PES. Fig. 4 shows that iNOS, TNF-α, and IL-6 mRNA levels were significantly reduced at 1 and 5 mg/kg of PES treatment, compared to LPS alone, in the liver. These results clearly demonstrate that inhibition of Hsp70 substrate binding activity prevents the induction of pro-inflammatory factors in LPS-stimulated liver at both protein and mRNA levels.

### PES suppresses the IκB-α-NF-κB pathway in liver

**Figure 5:**
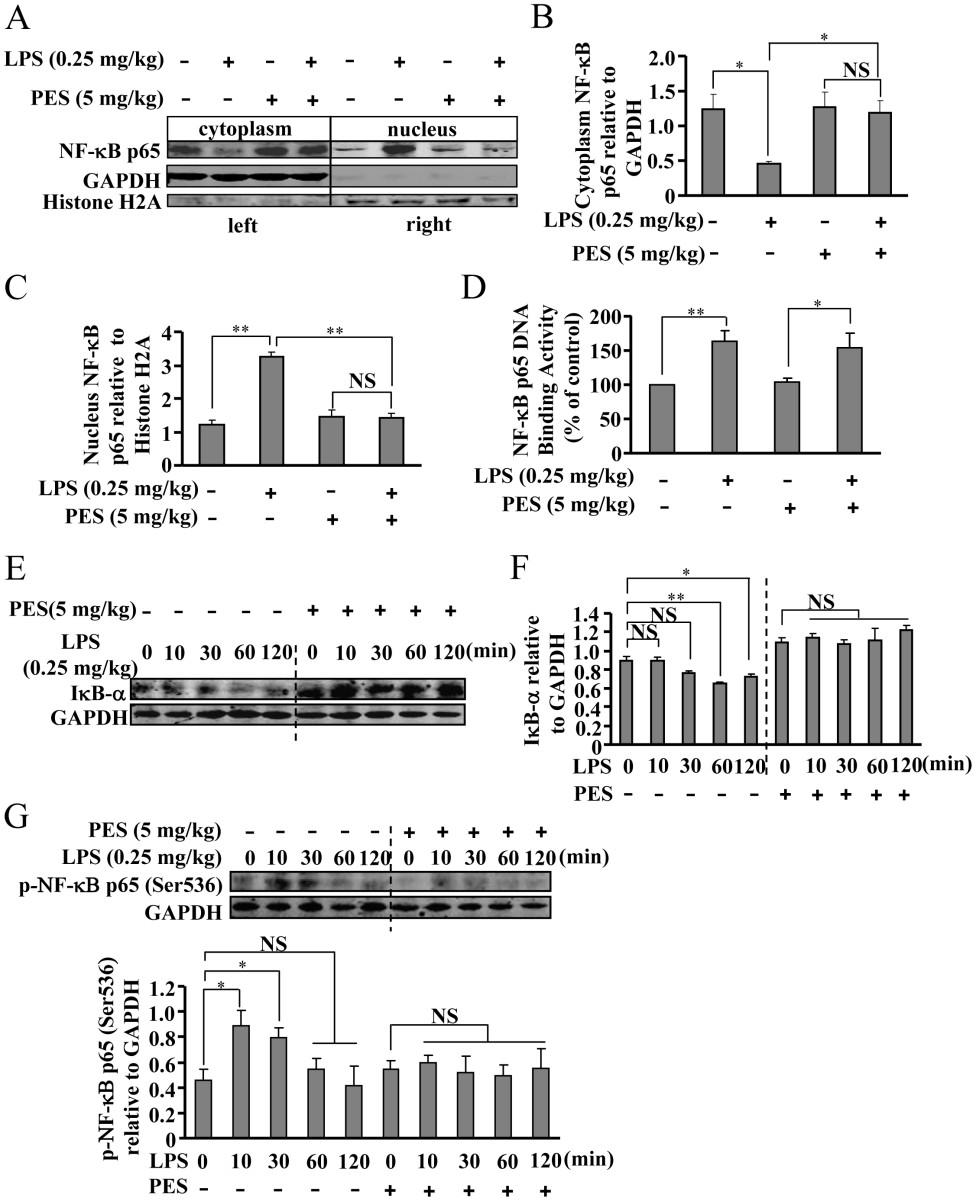
PES inhibits NF-κB nuclear translocation, IκB-α degradation, and NF-κB phosphorylation in LPS-stimulated liver. (A) Pretreatment with PES (5 mg/kg) significantly reduced NF-κB nuclear translocation (left part: cytoplasm; right part: nucleus) in LPS-stimulated (0.25 mg/kg) liver. (B, C) Quantitative analysis of NF-κB expression in the cytoplasm (B) and nucleus (C) after PES administration, n = 8, *P<0.05, **P<0.01 vs. control or PES alone group. (D) Quantitative analysis of the effect of PES on NF-κB binding to its promoter DNA in liver, n = 6, *P<0.05, **P<0.01 vs. control or PES alone-treated group. (E) Representative images showing that PES suppressed IκB-α degradation in LPS-stimulated liver. (F) Quantitative analysis of the effect of PES (5 mg/kg) on IκB-α degradation in LPS-stimulated liver, n = 8, *P<0.05, **P<0.01 vs. control mice. (G) Upper: representative images showing that PES down-regulated LPS-induced NF-κB phosphorylation level. Lower: quantitative analysis of the effect of PES on LPS-induced NF-κB phosphorylation in liver, n = 8, *P<0.05, vs. control mice. All data were shown as mean ± SE. NS: no significance.

To initiate gene transcription, active NF-κB must enter nuclei. To investigate whether PES treatment could influence the nuclear transport of NF-κB *in vivo*, we monitored the change in NF-κB level in the cytoplasm and nucleus from livers stimulated with LPS and/or PES. As shown in Fig. 5A, NF-κB was present predominantly in the cytoplasm in un-stimulated livers. LPS injection resulted in NF-κB nuclear translocation, and this translocation was significantly inhibited by PES treatment (Fig. 5B, 5C). To analyze the effect of PES upstream of NF-κB nuclear translocation, we tested the changes in IκB-α degradation and NF-κB phosphorylation in the liver after PES administration. Fig. 5E, 5F, and 5G show that PES at 5 mg/kg BW suppressed IκB-α degradation and NF-κB phosphorylation increase in LPS-stimulated livers, suggesting that PES suppresses NF-κB nuclear translocation via deactivation of the IκB-α-NF-κB pathway in the liver.

### PES reduces iNOS induction in LPS-stimulated macrophages

**Figure 6:**
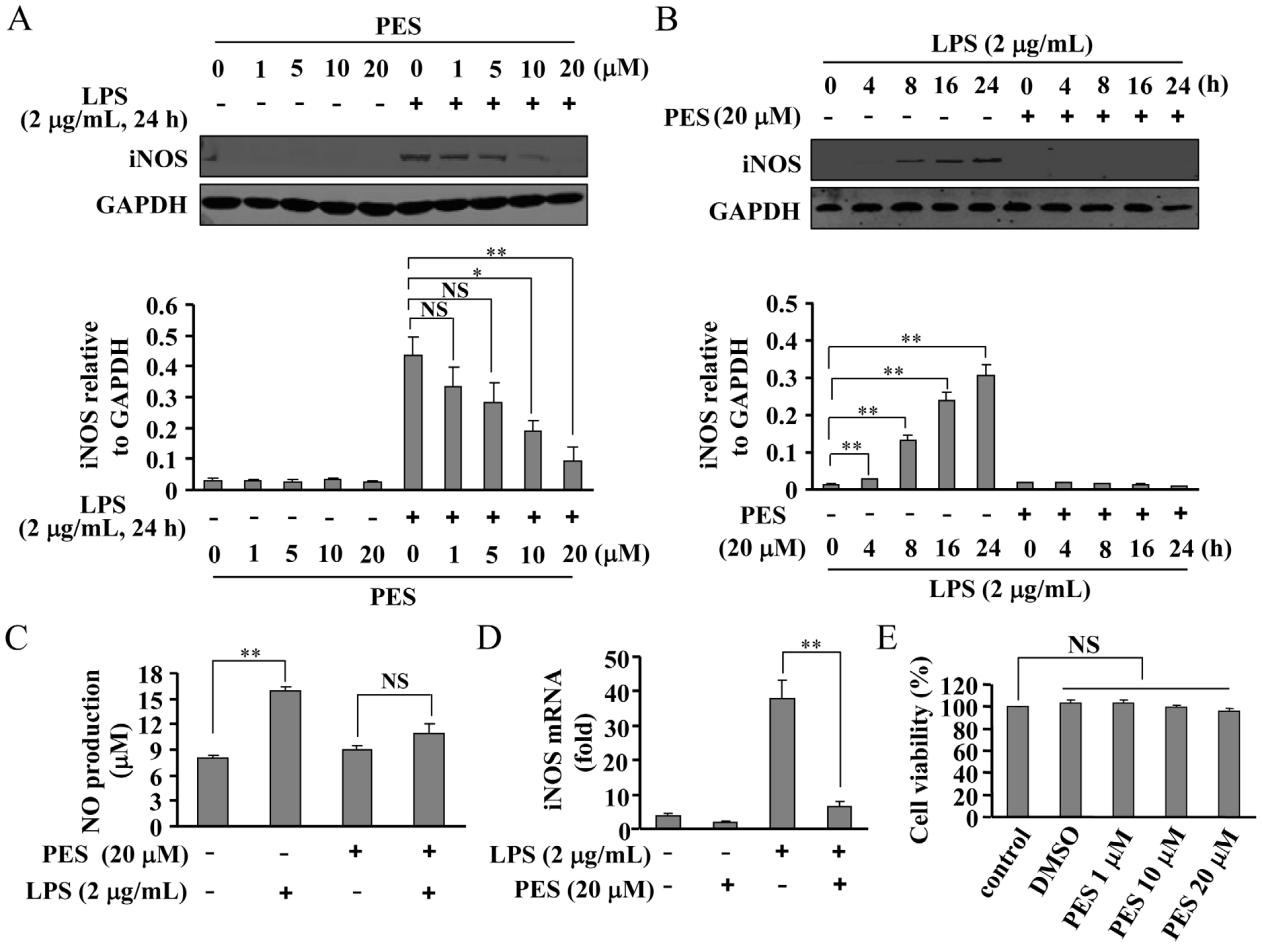
PES attenuates iNOS induction in both protein and mRNA levels in LPS-stimulated macrophages. (A) Upper: PES pretreatment (30 min) inhibited iNOS expression in LPS (2 µg/mL)-stimulated cells at different concentrations (1, 5, 10, 20 µM). Lower: quantitative analysis of iNOS expression in cells upon PES and/or LPS treatment (n = 3, *P<0.05, **P<0.01 vs. LPS alone treated-controls). (B) Upper: representative images showing the time-dependent (4, 8, 16, 24 h) effect of PES on LPS-induced iNOS expression. Lower: a time-course analysis of iNOS expression upon PES incubation (n = 3, **P<0.01 vs. control). (C, D) Quantitative analysis of NO content (C) and iNOS gene expression (D) in LPS- and/or PES-stimulated macrophages (n = 3, **P<0.01 vs. control). (E) Quantitative analysis of the cell viability of macrophages after PES (1, 10, and 20 µM) treatment (n = 8). All data were shown as mean ± S.E. NS: no significance.

To further explore the mechanisms by which PES influences the inflammatory response, RAW 264.7 cells, a well-accepted model for inflammation study, were employed in the following experiments. First, iNOS level was measured at different time points in response to varying concentrations of PES. RAW 264.7 cells were pre-treated with PES at concentrations ranging from 1–20 µM or vehicle for 30 min. Fig. 6A shows that PES induced a significant reduction in iNOS expression in macrophages. A peak change was observed at 20 µM PES. Cell viability was not affected by PES administered at 1, 10, and 20 µM. Thus 20 µM PES was selected for use in the following experiments. A time-dependent response curve shows that pretreatment of macrophages with PES (30 min) completely suppressed LPS-induced iNOS expression at 4, 8, 16, and 24 h time points (Fig. 6B). Secondly, NO content was detected. As shown in Fig. 6C, PES at 20 µM robustly reduced the NO content in LPS-stimulated macrophages. Finally, we found that iNOS mRNA level was significantly reduced by PES treatment in LPS-stimulated macrophages (Fig. 6D).

### PES suppresses IκB-α degradation and NF-κB activation, but not NF-κB DNA binding activity in macrophages

**Figure 7:**
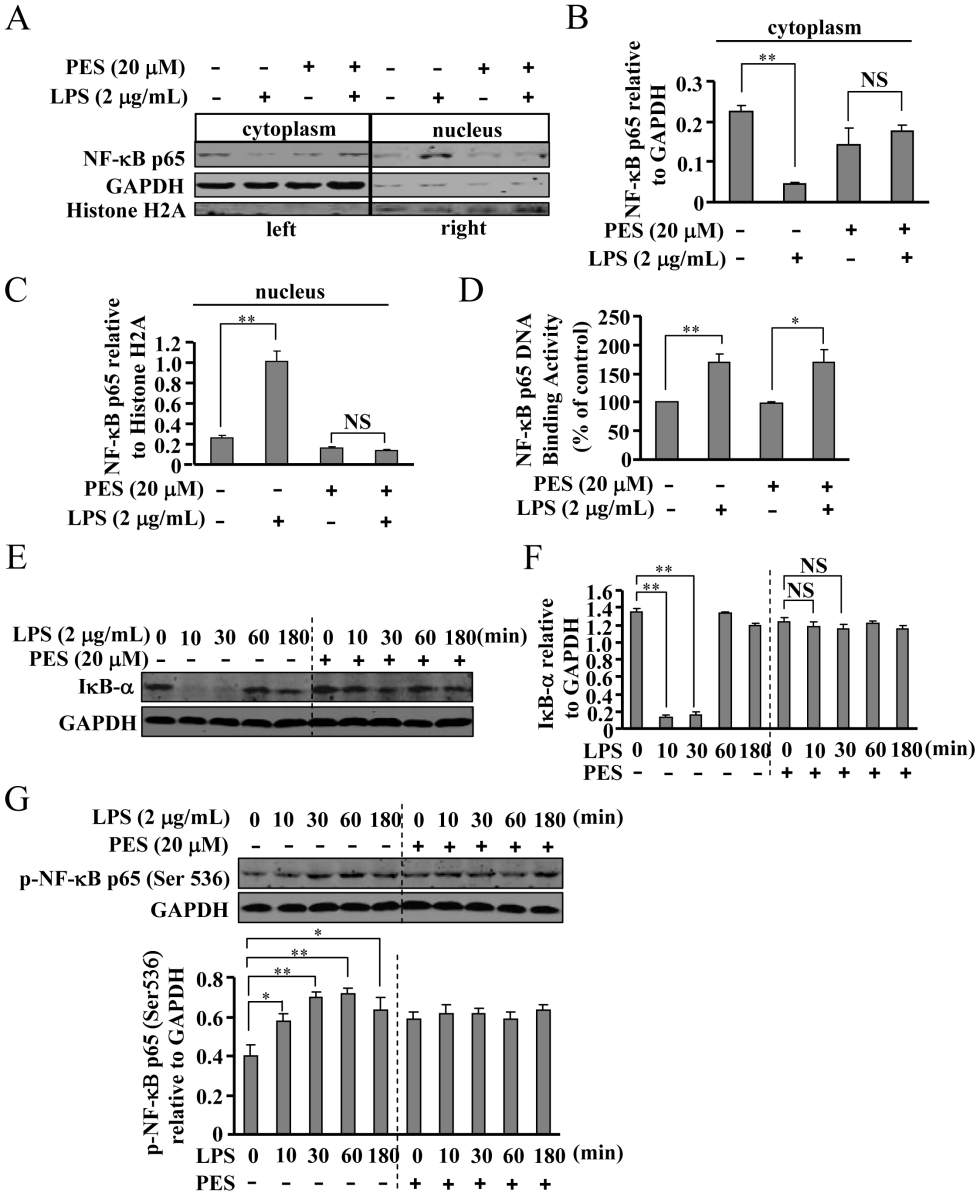
PES inhibits NF-κB nuclear translocation, IκB-α degradation, and NF-κB phosphorylation in LPS-stimulated macrophages. (A) Pretreatment with PES (20 µM) significantly reduced NF-κB nuclear translocation (left part: cytoplasm; right part: nucleus) in LPS (2 µg/mL)-stimulated macrophages. (B, C) Quantitative analysis of NF-κB expression in the cytoplasm and nucleus after PES administration, n = 3, **P<0.01 vs. control or PES alone-treated group. (D) Quantitative analysis of the effect of PES on NF-κB binding to its promoter DNA in macrophage, n = 3, *P<0.05, **P<0.01 vs. control or PES alone-treated group. (E) Representative images showing that PES suppressed IκB-α degradation in LPS-stimulated macrophages. (F) Quantitative analysis of the effect of PES on IκB-α degradation in LPS-stimulated macrophage, n = 3, **P<0.01 vs. control. (G) Upper: PES (20 µM) reduced the LPS (2 µg/mL)-induced NF-κB phosphorylation level. Lower: quantitative analysis of the effect of PES on LPS-induced NF-κB phosphorylation, n = 3, *P<0.05, **P<0.01 vs. control. All data were shown as mean ± SE. NS: no significance.

Next, we monitored the activation of IκB-α-NF-κB pathway in macrophages after PES treatment. As shown in Fig. 7A, 7B, and 7C, LPS-induced nuclear translocation of NF-κB was significantly attenuated by PES treatment. Similar to the liver, LPS also resulted in a significant degradation of IκB-α in macrophages, and pretreatment of cells with PES (20 µM, 30 min) markedly suppressed this degradation (Fig. 7E, 7F). In addition, we also observed a significant reduction in NF-κB phosphorylation level after PES treatment (20 µM) in LPS-stimulated macrophages (Fig. 7G). Besides the nuclear translocation of NF-κB, the binding activity of NF-κB to its DNA element is also important in mediating the gene transcription of pro-inflammatory factors. We thus determined whether the attenuation of NF-κB DNA binding activity contributes to the reduction in iNOS expression in macrophages after PES treatment. To explore this possibility, we stimulated macrophages with LPS (2 µg/mL, 10 min) to activate NF-κB. Then we measured the binding of active NF-κB with labeled DNA oligos corresponding to its promoter, in the absence or presence of PES. As shown in Fig. 7D, the NF-κB DNA binding activities in the nuclear fraction of lysates from LPS alone-treated macrophages were similar to that from LPS and PES-co-treated cells, demonstrating that the reduction in the production of pro-inflammatory factors is not associated with the change in nuclei NF-κB DNA binding activity in macrophages. Interestingly, the binding activity of NF-κB in liver was also not affected by PES administration (Fig. 5D).

### PES reduces the increase in [Ca^2+^]_i_ and pH_i_ in LPS-induced macrophages

**Figure 8:**
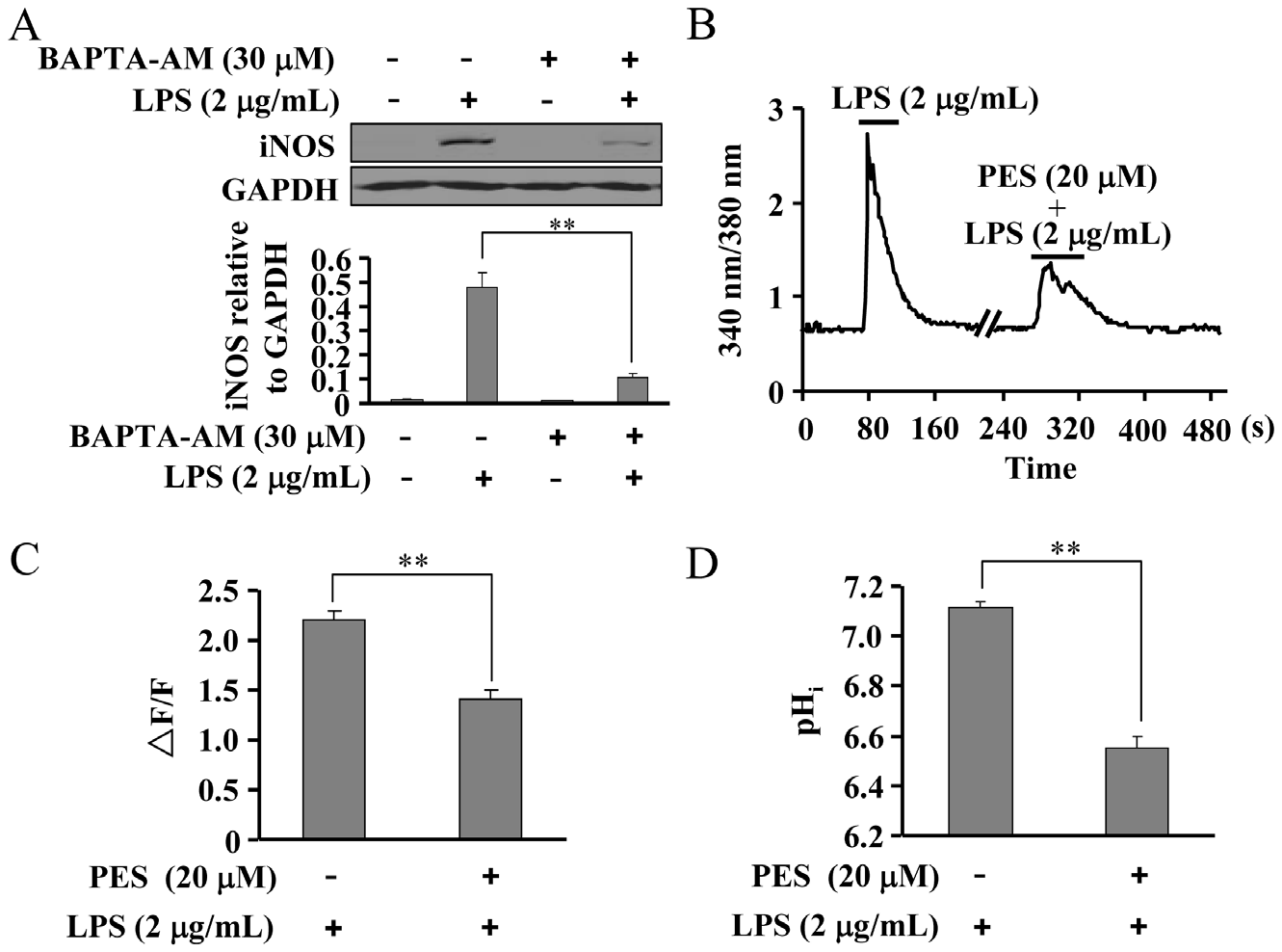
PES reduces the increase in [Ca^2+^]_i_ and intracellular pH value (pH_i_) in LPS-stimulated macrophages. (A) Upper: the effect of BAPTA-AM (20 µM) on iNOS expression in LPS (2 µg/mL)-stimulated macrophages. Lower: quantitative analysis of iNOS expression in macrophages pre-incubated with PES and treated with LPS. n = 3, **P<0.01 vs. LPS alone-treated group. (B) Representative 340/380 nm ratio of macrophages showing the change of [Ca^2+^]_i_ in macrophages as induced by LPS (2 µg/mL) in the absence or presence of PES (20 µM). (C) Summary data (▵F/F) of LPS-induced [Ca^2+^]_i_ elevation, n = 25 for LPS group; n = 30 for LPS and PES-co-treated group, **P<0.01 vs. control. (D) Summary data (▵F/F) of the change in pH_i_ of macrophages after LPS stimulation in the absence or presence of PES (20 µM). n = 28, **P<0.01 vs. control. All data were shown as mean ± SE.

[Ca^2+^]_i_ increase is considered to mediate the degradation of IκB-α and the subsequent activation of NF-κB [24], [25]. Here, we found that pretreatment of macrophages with BAPTA-AM (20 µM, 30 min), a chelator of intracellular Ca^2+^, significantly reduced iNOS expression in LPS-stimulated macrophage (Fig. 8A). This indicates that in this context, intracellular Ca^2+^ participates in the induction of pro-inflammatory factors. Further experiments showed that the increase in [Ca^2+^]_i_ in macrophages was remarkably reduced by PES treatment compared to LPS alone, (Fig. 8B, 8C), suggesting that inhibition of Hsp70 substrate binding activity might attenuate the induction of pro-inflammatory factors possibly by blocking the increase in [Ca^2+^]_i_ in LPS-induced macrophages. LPS was previously found to stimulate the increase in [H^+^]_i_ in macrophages, and this increase can be reversed by membrane NHE1 activity, ultimately resulting in an increase in pH_i_ and [Ca^2+^]_i_. We thus determined whether the effect of PES on [Ca^2+^]_i_ in macrophages was mediated by the change in pH_i_. As anticipated, pretreatment of macrophages with PES was found to significantly reduce the increase in pH_i_ in LPS-stimulated macrophages (Fig. 8D), indicating that Hsp70 substrate binding activity is required for the sensing of NHE1 to intracellular H^+^.

### PES disrupts NHE1-Hsp70 association in macrophages and liver

**Figure 9:**
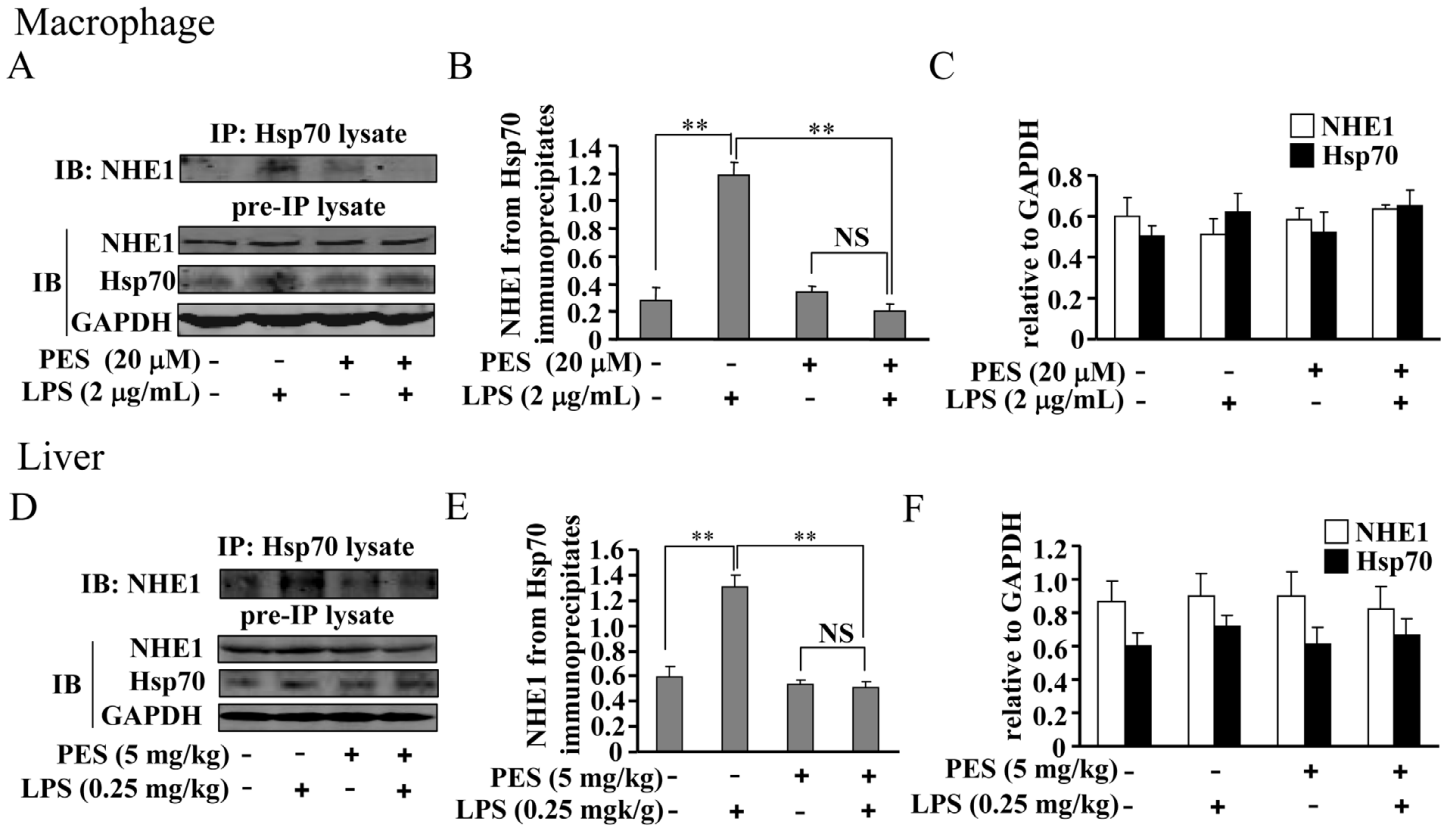
PES prevents NHE1 association to Hsp70 in LPS-stimulated macrophages. (A) Representative images showing that PES (20 µM) suppressed NHE1-Hsp70 association in LPS (2 µg/mL)-stimulated macrophages. (B) Quantitative analysis of NHE1-Hsp70 association in LPS- and/or PES-treated macrophages, n = 3, **P<0.01 vs. control or PES alone group. (C) Summary data showing the effect of PES on NHE1 and Hsp70 expression in macrophages (n = 3). (D) Representative images showing that PES (5 mg/kg) suppressed the association of NHE1 to Hsp70 in LPS (0.25 mg/kg)-stimulated liver tissues. (E) Quantitative analysis of NHE1 and Hsp70 association in LPS- and/or PES-stimulated liver tissues, n = 6, **P<0.01 vs. control or PES alone group. (F) Summary data showing the effect of PES on NHE1 and Hsp70 expression in liver (n = 6). All data were shown as mean ± SE. NS: no significance.

**Figure 10:**
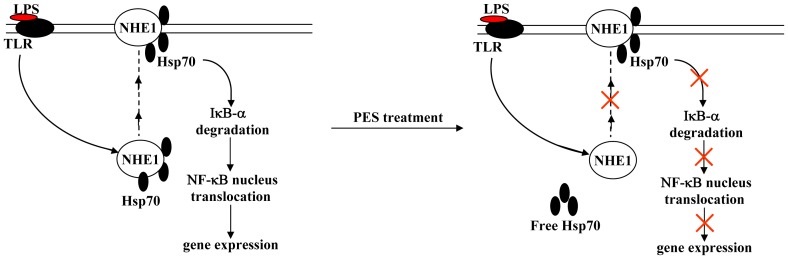
Proposed scheme for the influence of PES on the signaling pathways downstream of LPS-induced inflammatory gene expression in macrophages.

How exactly Hsp70 assists in the sensing of NHE1 to intracellular H^+^ is still elusive. Here we found that LPS robustly enhanced the NHE1 association to Hsp70 in cultured macrophages, and this enhancement was significantly reduced by 20 µM PES compared to LPS alone (Fig. 9A). In addition, we observed that Hsp70 expression was slightly induced by LPS treatment in macrophages, and PES treatment had no significant effect on LPS-induced Hsp70 expression (Fig. 9B, 9C). These data indicate that the increase in NHE1-Hsp70 association might mediate the LPS-induced induction of pro-inflammatory factors, and PES exerts inhibitory functions likely by disrupting the NHE1-Hsp70 complex (Fig. 10). We also measured the association between NHE1 and Hsp70 in whole liver tissues. Intriguingly, our results showed that PES treatment also completely reduced the increase in NHE1 association to Hsp70 in LPS-stimulated liver (Fig. 9D, 9E, 9F), suggesting that PES suppresses the inflammatory responses *in vivo* by reducing the NHE1-Hsp70 association.

## Discussion

Intracellular chaperones are essential for the stability and function of signaling molecules downstream of the LPS receptor [26], [27]. The role of Hsp70, an important molecular chaperone in the LPS signaling pathway, has been recognized [5], [6], [7]. The significance of LPS-mediated macrophage activation and inflammatory response in acute liver injury is well studied [28]. In the present study, we targeted Hsp70 substrate binding activity to inhibit LPS signaling in the liver and reduce induction of pro-inflammatory factors in order to ameliorate liver injury. Through experiments performed *in vivo* using PES, an Hsp70 substrate binding activity inhibitor, we showed that inhibition of Hsp70 substrate binding activity decreases the infiltration of inflammatory cells as well as the induction of pro-inflammatory factors in liver, and alleviates LPS-induced liver injury. Our results here suggest that inhibition of Hsp70 substrate binding activity is a novel therapeutic approach in treating liver inflammatory disorders.

Although inhibition of Hsp70 substrate binding activity by PES has been demonstrated to be of potential therapeutic importance in lymphoma [8] and leukemia [9], its effect on inflammatory disorders is still elusive. Because LPS-mediated acute inflammatory response contributes greatly to the progression of liver disorders, strategies that ameliorate this response in the liver could have a beneficial effect. In fact, in our present study, LPS-mediated inflammatory responses and liver injury were successfully prevented by PES, suggesting that PES might be a useful agent to protect liver from inflammatory injury. The role of PES in decreasing LPS-mediated induction of inflammatory factors in liver and macrophages could be due to its ability to inhibit the canonical inflammatory response pathway. Propagation of inflammation occurs through signaling from the stimuli to the corresponding transcriptional factor, in which IκB-α removal often occurs after stimuli application [29], [30]. IκB-α degradation was observed in both LPS-stimulated cells and liver tissues, and this was inhibited by PES treatment. NF-κB phosphorylation and nuclear translocation lies downstream of IκB-α degradation [31]. NF-κB was retained in the cytoplasm at resting state, and this retention is mediated by IκB family inhibitory proteins which can mask the nuclear localization sequence of NF-κB [32]. Upon activation, NF-κB is transported into nucleus, where it binds to its DNA element in order to regulate gene expression [31]. Here we found that the inhibitory effects of PES on LPS-induced induction of pro-inflammatory factors were mediated through the attenuation of NF-κB nuclear translocation but not disruption of DNA binding activity in both macrophages and liver. In general, we conclude that the LPS-IκB-α-NF-κB pathway mediates the attenuation of induction of pro-inflammatory factors observed upon inhibition of Hsp70 substrate binding activity in liver.

How exactly the Hsp70 substrate binding activity is involved in LPS signaling is not clear. Earlier data have shown that LPS activates membrane NHE-1 to elevate internal Ca^2+^ for IκB-α degradation and NF-κB activation in both microglia and macrophages [16], [20], [24], [25], [33]. Our present data also showed that PES reduced the increase in pH_i_ as well as the increase in internal Ca^2+^ after LPS stimulation in RAW 264.7 cells, indicating that attenuation in NHE1 activation is involved in the inhibition of PES on the induction of inflammatory response in macrophages. A novel function of NHE-1 is to integrate signaling information by acting as a scaffolding platform [17], [34]. This platform facilitates signal relay through different signaling pathways. In this study, we observed a remarkably increase in NHE1 association to Hsp70 after LPS treatment in both macrophages and liver, demonstrating that the formation of NHE1-Hsp70 complex is essential for the induction of pro-inflammatory factors. This finding about the role of PES in NHE1-Hsp70 association, together with the effects of PES on the induction of pro-inflammatory factors intriguingly reveals how pro-inflammatory cells initiates the sensing of NHE1 to intracellular H^+^ after inflammatory stimulation, and how this process needs the NHE1-Hsp70 interaction during signaling transduction. However, since RAW 264.7 cells do not exactly behave as the inflammatory cells such as Kupffer cells and neutrophilic granulocytes in liver, these conclusions, to some extent, limit our observations for the role of PES in liver injury. Experiments in different inflammatory cells need to be further examined. In addition, there still remains a question: how LPS/TLR4 signaling influences NHE1-Hsp70 complex and interaction to promote inflammatory responses. A previous study reported that PES also inhibited the TNF-α-induced degradation of IκB-α in H1299 cells [8], indicating that molecules upstream of IκB-α might be involved in the function of PES in inflammation. Data from Agou et al cued that the IκB kinase gamma (IKKγ, NEMO) might be a potential candidate for this hypothesis [35]. Our results provide further evidence (data not shown): i. LPS increased the association of NEMO to Hsp70 in cultured macrophages, which is in line with the fact that NEMO associates with Hsp70 via its coiled-coil C-terminal domain [35], [36]; ii. PES significantly reduced this association. This result showed that Hsp70 may play a key role in controlling the biological activity of NEMO and thereby in the activation of NF-κB or in the inhibition of NF-κB by PES through modulation of NEMO-Hsp70 interaction. Experiments attempting to correlate the increase in NEMO-Hsp70 interaction with the increase in NHE1-Hsp70 interaction in LPS/TLR4 signaling are in progress.

Overexpression of Hsp70 was previously found to inhibit induction of pro-inflammatory cytokines in both macrophages [5] and microglia [6]. The similarity between the effects of Hsp70 overexpression and Hsp70 substrate binding activity inhibition on the induction of pro-inflammatory factors suggests that increasing Hsp70 expression and inhibiting Hsp70 activity might be beneficial in treating inflammatory disorders. However, it is worth noting that overexpression of Hsp70 does not always exert protective functions. Recombinant Hsp70 was found to trigger a cytotoxic response in T-helper cells [37], and high expression of cell-surface Hsp70 and high serum levels of circulating Hsp70 correlate with a shorter survival for AML patients [38]. Thus the biological effects of Hsp70 expression are more complicated than that of Hsp70 activity. Due to the accessibility of small artificial molecules, regulating Hsp70 activity would be more promising in the future exploitation of therapeutic agents for disorders other than inflammation. In fact, numerous studies have already given us plenty of evidence to support this supposition. For example, PES was found to possess anti-leukemic actions in human primary AML blasts [9]. Repression of Hsp90 activity prevents LPS-induced iNOS expression [39] and liver injury [21]. More importantly, one of the Hsp70 ATPase inhibitors, methylene blue, has also entered phase III trials to treat Alzhaimer’s disease patients [40].

In conclusion, our study shows a novel role for an Hsp70 substrate binding activity inhibitor in alleviating LPS-mediated liver injury, thus providing a potential application of Hsp70 substrate binding activity inhibitors in liver inflammatory disorders therapies. Mostly importantly, our results, which focused on the effect of PES on the interaction between NHE1 and Hsp70 in liver, demonstrate for the first time the indispensable role of NHE1-Hsp70 association in the induction of inflammatory response (Fig. 10).
